# Association between antidepressant use and liver fibrosis in patients with type 2 diabetes: a population based study

**DOI:** 10.1186/s13098-023-01016-x

**Published:** 2023-03-11

**Authors:** Lin Shi, Fangyuan Jia

**Affiliations:** Department of Gastroenterology, Xuzhou Central Hospital, Xuzhou Clinical School of Xuzhou Medical University, Xuzhou, Jiangsu China

**Keywords:** Antidepressant use, Liver fibrosis, NAFLD, Diabetes

## Abstract

**Background:**

The prevalence of liver fibrosis among diabetic patients is increasing rapidly. Our study aims at exploring the relationship between antidepressant use and liver fibrosis in diabetic patients.

**Methods:**

We conducted this cross-sectional study through the cycle of National Health and Nutrition Examination Survey (NHANES) 2017–2018. The study population were consisted of patients with type 2 diabetes and reliable vibration-controlled transient elastography (VCTE) results. The presence of liver fibrosis and steatosis were assessed by the median values of liver stiffness measurement (LSM) and controlled attenuation parameter (CAP), respectively. Antidepressants included selective serotonin reuptake inhibitors (SSRIs), tricyclic antidepressants (TCAs), serotonin and norepinephrine reuptake inhibitors (SNRIs) and serotonin antagonists and reuptake inhibitors (SARIs). Patients with evidence of viral hepatitis and significant alcohol consumption were excluded. Logistic regression analysis was performed to evaluate the association between antidepressant use and both steatosis and significant (≥ F3) liver fibrosis after adjustment for potential confounders.

**Results:**

Our study population consisted of 340 women and 414 men, of whom 87 women(61.3%) and 55(38.7%) men received antidepressants. The most commonly used antidepressants were SSNIs(48.6%), SNRIs(22.5%) and TCAs(12.7%), followed by SARIs(10.6%) and other antidepressants(5.6%). 165 participants had significant liver fibrosis by VCTE, with a weighted overall prevalence of 24%(95% CI 19.2–29.5). In addition, 510 patients had evidence of hepatic steatosis by VCTE with a weighted overall prevalence of 75.4%(95% CI 69.2–80.7). After adjusting confounders, no significant association was observed between antidepressant use and significant liver fibrosis or cirrhosis.

**Conclusions:**

In conclusion, in this cross-sectional study, we found that antidepressant drugs was not associated with liver fibrosis and cirrhosis in patients with type 2 diabetes in a nationwide population.

## Introduction

The global prevalence and incidence of diabetes and mental health problems are increasing rapidly, especially the high risk of depression symptoms for diabetic patients compared to those without diabetes [1, 2]. Comorbid diabetes and depression are associated with increased mortality, such as cardiovascular or kidney disease [3, 4]. Moreover, the coexistence of diabetes and depression has a greater impact on depression when combined with other diseases [2]. On the other hand, non-alcoholic fatty liver disease (NAFLD) is very common among diabetic patients. It was reported that 60–70% of patients with type 2 diabetes(T2DM) have non-alcoholic fatty liver disease (NAFLD), and nearly 15% have evidence of advanced liver fibrosis [5, 6]. In addition, a study of a nationally representative sample of US adults showed that depression was independently associated with the occurrence of NAFLD [7]. Therefore, patients with T2DM are at a high risk of combinational NAFLD or liver fibrosis and depression, and the complex interplay of those diseases is not fully understood.

Depression disorders among patients with T2DM could be effectively treated by antidepressants, as has been reported in some studies [2, 8]. However, some recent studies have shown that antidepressant use might be a risk factor for the new onset of T2DM, especially tricyclic antidepressant use [9, 10]. Moreover, an increasing linear relationship between the duration of antidepressant use and T2DM risk was observed in a recent meta-analysis [9].In addition, NAFLD is common in T2DM, and patients with T2DM suffer from an increased risk of liver mortality. In patients with viral hepatitis, antidepressants were considered to play a protective role in liver fibrosis progression and reduce the risk of developing cirrhosis [11, 12].Considering the increased prevalence of depression disorders, antidepressant use and NAFLD among T2DM patients, understanding the relationship between antidepressant use and liver fibrosis in patients with type 2 diabetes is critical.

In fact, few studies focused on the relationship between antidepressant use and liver fibrosis in patients with T2DM. In our study, we extracted data from an unselected sample of adults with T2DM from the 2017–2018 cycle of the National Health and Nutrition Examination Survey (NHANES) to examine the association between antidepressant use and liver fibrosis.

## Materials and methods

### Study population

This study was conducted on the basis of the National Health and Nutrition Examination Survey (NHANES) 2017–2018. The NHANES is conducted in the United States by the National Center for Health Statistics (NCHS) of the Centers for Disease Control and Prevention (CDC). All the data in the NHANES were collected by unified trained professional personnel through household interviews or an examination conducted in a mobile examination center. The survey consists of cross-sectional interviews, examinations and laboratory data collected from a complex multistage, stratified, clustered probability sample representative of the US population. The Institutional Review Board of the CDC approved the survey protocol. All participants provided informed consent. We were exempt from IRB approval from our institution as the dataset used in the analysis was completely deidentified. The participants were adults aged 18 years or older with type 2 diabetes in 2017–2018. In our analysis, the exclusion criteria were as follows: (1) participants with positive serum hepatitis B surface antigen or positive serum hepatitis C antibody; (2) participants with significant alcohol consumption(≥ 20 g/day for men and ≥ 10 g/day for women); (3) participants without complete vibration controlled transient elastography(VCTE); and (4) participants with possible type 1 diabetes(defined as a diagnosis at age < 30 years and the use of insulin as the only anti-diabetic therapy) [13].

### Clinical and laboratory variables

A series of potential confounders in the association of antidepressant use and liver fibrosis were extracted from the NHANES database, including demographic, clinical and laboratory data. Demographic variables included age, sex, ethnicity(Hispanic, non-Hispanic white, non-Hispanic black, or other races), the ratio of family income to poverty threshold, smoking habits and alcohol habits. Body measurements such as body mass index (BMI) and blood pressure were included. Past medical history, including ever diagnosis and treatment of coronary heart disease, stroke and hypertension was also included. The diagnosis of diabetes was based on any of the following criteria: (1) A self-reported diagnosis of diabetes. (2) use of anti-diabetic drugs. (3) a hemoglobin A1c (HbA1c) level ≥ 6.5% (48 mmol/mol) [13].

Laboratory variables included alanine aminotransferase (ALT), aspartate aminotransferase (AST), γ-glutamyltranspeptidase (GGT), total bilirubin(TBIL), glycated hemoglobin(HbA1c), total cholesterol, high density lipoprotein (HDL) and cholesterol. Laboratory methods for measurements of these values were reported in detail elsewhere [13, 14].

### Antidepressant exposure and liver fibrosis assessment

During the in-home questionnaire, trained interviewers reviewed participants' pill bottles for prescription and nonprescription medications and supplements reported to have been taken in the previous month [15]. Selective serotonin reuptake inhibitors (SSRIs) included escitalopram, fluoxetine, citalopram, fluvoxamine, sertraline, paroxetine, and duloxetine. Tricyclic antidepressant (TCA) prescriptions included amoxapine, amitriptyline, protriptyline, nortriptyline, imipramine, desipramine, doxepin, and trimipramine. Serotonin and norepinephrine reuptake inhibitors (SNRIs) included duloxetine and venlafaxine. Serotonin antagonists and reuptake inhibitors (SARIs) included trazodone and buspirone. Other antidepressants included mirtazapine and bupropion. The interviews, data collection and data processing methods were performed under the NHANES protocol.

Vibration controlled transient elastography(VCTE) was used and validated in some previous studies concerning liver fibrosis assessment in participants with nonalcoholic fatty liver disease. The detailed methods and steps of VCTE have been reported elsewhere [16, 17]. In our present study, we only included participants with complete VCTE assessment, which has been reported elsewhere [13]. For our analysis, we defined liver stiffness measurement(LSM) values of 8.0 kPa [18, 19] or higher and 13.1 kPa [19] as significant fibrosis (≥ F2) and cirrhosis (≥ F4) according to previous studies. Moreover, participants who had controlled attenuation parameter(CAP) values of 274 dB/m or higher and 302 dB/m were considered to have steatosis (≥ S1) and severe steatosis (≥ S3) [13].

### Statistical analysis

The PASS software (version 11) was used to calculate sample size. As a cross-sectional study, the overall prevalence of depression among diabetic patients was approximately 20% in previous studies [9]. Therefore, 20% was set as the estimated prevalence of depression among diabetic patients. The allowable error of the overall estimated proportion was set as 0.03. The significance level was set at 0.05, and a two-sided interval was used. Based on PASS software, the sample size was calculated as 715, which is lower than that in our study [20].

All analyses were conducted by R software(4.1.1). Due to the complex design of the NHANES survey, we followed the recommendations of the NHANES and a two-year-cycle weights were appropriately used in our study for each analysis. Categorical variables are expressed as numbers and weighted proportions. Continuous variables are presented as weighted means ± standard error (SE).

All enrolled participants were separated into two groups: antidepressant use group and no-antidepressant use group. The characteristics between two groups were compared by sample weighted linear regression for continuous variables and the design-adjusted Rao-Scott chi-square test for categorical variables. To further examine the association between antidepressant exposure and liver fibrosis, we applied two weighted multivariable linear regression analyses. In Model 1,we only adjusted for covariates including age, sex and race. In Model 2, we further adjusted Model 1 plus BMI and laboratory variables including ALT, AST, GGT, triglycerides and HbA1c. The relationship between antidepressant use and liver steatosis was also examined using the two models. A two-tailed P value < 0.05 was considered statistically significant.

## Results

### Baseline characteristics of the study population

**Fig. 1:**
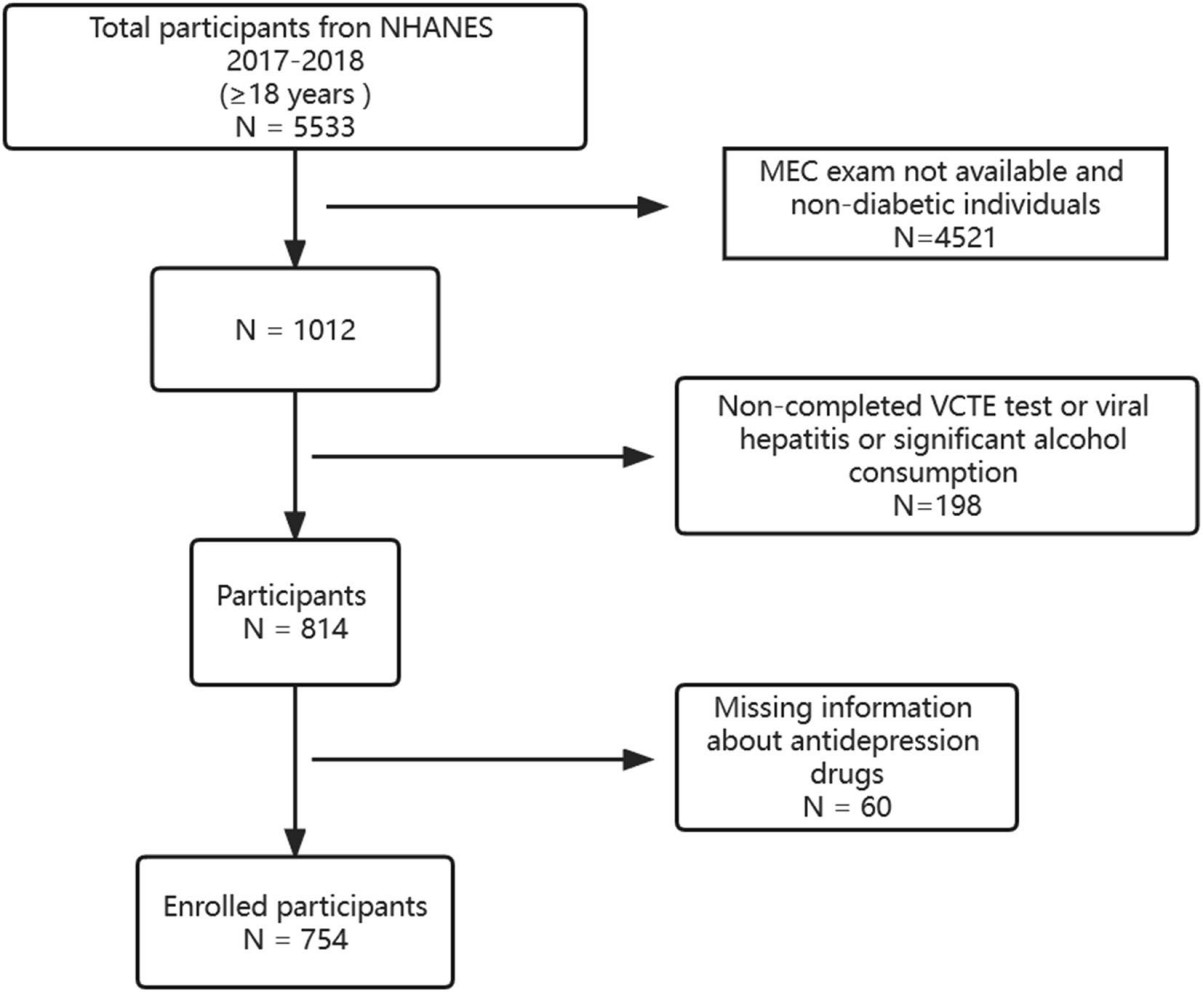
The flow chart of our study

A total of 5533 individuals aged over 18 years with complete MEC visits during interviews in the2017-2018 NHANES cycle were identified as initial samples. Then we excluded participants without diabetes or type 1 diabetes and viral hepatitis and significant alcohol intake as previously described. Finally, we enrolled participants with complete VCTE data, leading to a population of 754 patients as our final study population. The flow chart of our study is shown in Fig. 1.

Our study population comprised of 340 women and 414 men, of whom 87 women (61.3%) and 55 (38.7%) men received antidepressants. A significantly higher BMI was observed in the antidepressant use group (33.6 ± 7.4 vs. 31.8 ± 7.5). In our study, 14.5% of patients were lean (BMI < 25 kg/m2), 29.7% were overweight (BMI 25–29.9 kg/m2) and 55.8% were obese (BMI ≥ 30 kg/m2). Women more frequently received antidepressants than men. The most commonly used antidepressants were SSNIs (48.6%), SNRIs (22.5%) and TCAs (12.7%), followed by SARIs (10.6%) and other antidepressants (5.6%). A total of 165 participants had significant liver fibrosis by VCTE for an overall weighted prevalence of 24% (95% CI 19.2–29.5). Moreover, 510 patients had evidence of liver steatosis by VCTE with an overall weighted prevalence of 75.4% (95% CI 69.2–80.7).

We compared the clinical and laboratory variables between the antidepressant use group and the non-antidepressant use group in Table 1. There was no significant in age between the two groups, and non-Hispanic White patients tended to received more antidepressants.

**Table 1:** Features of the study population according to current antidepressant use

	Non-antidepressant use	Antidepressant use	P-value
N	612	142	
Age(years)	61.9 ± 13.0	61.5 ± 13.4	0.806
Male(%)	359 (58.7%)	55 (38.7%)	< 0.001
Race/ethnicity (%)			< 0.001
Non-Hispanic white	171 (27.9%)	69 (48.6%)	
Non-Hispanic Black	135 (22.1%)	34 (23.9%)	
Mexican American	102 (16.7%)	11 (7.7%)	
other	204 (33.3%)	28 (19.7%)	
BMI(kg/m^2^)	31.8 ± 7.5	33.6 ± 7.4	0.003
Waist circumference (cm)	107.9 ± 16.2	111.4 ± 15.5	0.02
Laboratory variables			
ALT(IU/L)	24.5 ± 17.5	19.2 ± 9.9	< 0.001
AST(IU/L)	22.6 ± 14.2	19.7 ± 8.0	0.009
Albumin(g/L)	40.1 ± 3.1	38.7 ± 4.0	< 0.001
GGT(IU/L)	39.9 ± 48.4	34.7 ± 46.4	0.015
HDL(mg/dL)	48.7 ± 13.6	49.0 ± 14.6	0.965
LDL(mg/dl)	2.87 ± 0.04	2.73 ± 0.06	0.05
Total cholesterol (mg/dL)	179.4 ± 45.0	172.4 ± 45.8	0.019
Triglycerides (mg/dL)	135.0 ± 116.3	174.6 ± 178.2	0.032
HbA1c (%)	7.2 ± 1.6	7.1 ± 1.4	0.271
LSM(kPa)	7.2 ± 5.9	7.5 ± 9.0	0.391
CAP(dB/m)	302.1 ± 55.9	304.3 ± 55.7	0.752
Fibrosis≧2	139 (22.7%)	26 (18.3%)	0.253
Fibrosis≧4	43 (7.0%)	8 (5.6%)	0.552
Steatosis≧1	411 (67.2%)	99 (69.7%)	0.557
Steatosis≧3	315 (51.5%)	73 (51.4%)	0.989

*BMI* body mass index, *ALT* alanine transaminase, *AST* aspartate transaminase, *GGT* gamma-glutamyl transpeptidase, *HDL* high density lipoprotein, *LDL* low density lipoprotein, *HbA1c* glycosylated hemoglobin, *LSM* liver stiffness measurement, *CAP* controlled attenuation parameter;

Some liver function markers, including ALT, AST and TBIL, were significantly lower in the antidepressant use group. However, no significant differences were observed in CAP and LSM between the two groups. Interestingly, lipid parameters including LDL, total cholesterol and triglycerides, were significantly lower in the antidepressant use group, whereas HDL cholesterol was not significantly different between the two groups.

### Liver fibrosis and steatosis

To examine the association between liver fibrosis and antidepressant use, we then conducted sample weighted multivariable logistic regression models (Table 2). We also identified factors related to steatosis by above models (Table 3).

**Table 2:** Association of antidepressant with risk of significant fibrosis or cirrhosis by weighted regression

Variables	Significant fibrosis (≥ F2)			Cirrhosis (F4)		
	OR	95%CI	P-value	OR	95%CI	P-value
Sex	1.3	0.27–6.35	0.64	1.29	0.38–4.98	0.79
Age	1.01	0.99–1.04	0.34	1.01	0.98–1.05	0.68
Race	-	-	-	-	-	-
Non-Hispanic white	Ref	-	-	Ref	-	
Non-Hispanic Black	0.63	0.53–0.91	0.03	1.44	0.99–1.99	0.25
Mexican American	1.82	0.87–3.80	0.11	1.55	0.75–3.20	0.23
Other	1.95	0.21–3.13	0.19	1.75	0.98–2.77	0.2
BMI	1.14	1.1–1.18	0.001	1.17	1.08–1.26	0.01
ALT	0.99	0.94–1.04	0.99	0.87	0.79–1.96	0.21
AST	1.2	1.01–1.03	0.03	1.01	1.01–1.02	0.05
GGT	1.01	1.00–1.03	0.1	1.01	1.00–1.01	0.09
HbA1c	1.1	0.97–1.24	0.20	1.09	0.91–1.38	0.5
Triglycerides	1.02	1.01–1.04	0.04	0.99	0.99–1.00	0.99
LDL	1.08	0.73–1.6	0.68	0.98	0.96–1.01	0.19
Antidepressant use	0.53	0.2–1.58	0.31	0.49	0.07–2.41	0.51

*BMI* body mass index, *ALT* alanine transaminase, *AST* aspartate transaminase, *GGT* gamma-glutamyl transpeptidase, *LDL* low density lipoprotein, *HbA1c* glycosylated hemoglobin

**Table 3:** Association of antidepressant with risk of any or severe steatosis by weighted regression

Variables	Any steatosis (≥ S1)			Severe steatosis (S3)		
	OR	95%CI	P-value	OR	95%CI	P-value
Sex	1.51	0.88–2.36	0.29	1.81	1.03–3.01	0.04
Age	1.02	0.99–1.05	0.25	1.01	0.99–1.15	0.59
Race	–	–	–	–	–	–
Non-Hispanic white	Ref	–	–	Ref	–	–
Non-Hispanic Black	0.51	0.07–0.89	0.04	0.49	0.17–2.89	0.1
Mexican American	1.54	0.78–3.05	0.21	1.87	0.65–3.0	0.33
Other	1.77	1.0–2.82	0.61	1.58	0.77–2.98	0.79
BMI	1.15	1.07–1.22	0.01	1.11	1.00–1.20	0.06
ALT	1.02	1.01–1.04	0.03	1.03	1.01–1.08	0.05
AST	0.96	0.89–1.0	0.12	0.99	0.99–1.03	0.61
GGT	1.0	0.99–1.01	0.77	1.0	0.99–1.00	0.38
HbA1c	1.21	1.05–1.49	0.03	1.27	0.99–1.51	0.32
Triglycerides	1.01	1.0–1.02	< 0.001	1.01	1.0–1.02	< 0.001
LDL	0.87	0.71–1.07	0.18	0.81	0.71–1.11	0.19
Antidepressant use	0.79	0.41–1.84	0.39	0.87	0.39–1.55	0.59

*BMI* body mass index, *ALT* alanine transaminase, *AST* aspartate transaminase, *GGT* gamma-glutamyl transpeptidase, *LDL* low density lipoprotein, *HbA1c* glycosylated hemoglobin;

After adjusting for confounding factors, no significant association was observed between antidepressant use and significant liver fibrosis (OR: 0.53; 95% CI: 0.2–1.58) or cirrhosis (OR: 0.49; 95% CI: 0.07–2.41). In the analysis, we also identified that BMI was significantly associated with significant fibrosis (OR: 1.14; 95% CI: 1.1–1.18) or cirrhosis (OR: 1.17; 95% CI: 1.08–1.26). Higher triglyceride was also associated with fibrosis (OR: 1.02, 95% CI: 1.01–1.04) but not with cirrhosis (OR: 0.99, 95% CI: 0.99–1.00). Non-Hispanic black individuals were protected from liver fibrosis (OR: 0.63; 95% CI: 0.53–0.91) but not in cirrhosis (OR: 1.44; 95% CI: 0.99–1.99). Detailed regression results are displayed in Table 2.

Considering the severity of hepatic steatosis, BMI and HbA1c were independent risk factors or any steatosis (OR: 1.15, 95% CI: 1.07–1.22; OR: 1.21, 95% CI: 1.05–1.49, respectively) but were not associated with severe steatosis(OR: 1.11, 95% CI: 1.00–1.12; OR: 1.27, 95% CI: 0.99–1.51, respectively) (Table 3). Moreover, non-Hispanic black individuals were protected from steatosis (OR: 0.51; 95% CI: 0.07–0.89) but were not associated with severe steatosis(OR: 0.49; 95% CI: 0.17–2.89). Similarly, we found no significant association between antidepressant use and liver steatosis(OR: 0.87; 95% CI: 0.39–1.55). Other regression results are shown in Table 3.

## Disscussion

In the present study, we enrolled 754 patients and found that after adjusting for confounding factors, antidepressant use was not statistically associated with liver fibrosis and steatosis among patients with T2DM. In our study, we included several main types of antidepressant medications, such as SSNIs, SNRIs, and TCAs. Considering the increasing incidence of depression disorders in T2DM patients, our study improves the understanding of the association between antidepressant drugs and liver fibrosis in patients with type 2 diabetes.

Patients with T2DM have long been plagued with NAFLD and mental disorders according to data from the famous INTERPRET-DD study [1]. In that international and multicenter analysis, the researchers found that 10.6% of diabetic patients were diagnosed with current major depressive disorder, and 17% of diabetic patients reported moderate/severe depressive symptomatology. Thus, mental disorders are prevalent among diabetic patients. On the other hand, NAFLD has also been a huge burden on public health and affects most individuals with T2DM. Moreover, the incidence of NAFLD-induced liver fibrosis is increasing, which may have a negative impact on the health of patients with T2DM, which may have different impacts in different sexes, and public health systems [21–23]. To date, we have no effective drug therapies for NAFLD-induced liver fibrosis.

To date, only a few drugs have been explored and validated in slowing the progression of liver fibrosis. It has been reported that statins may play an important role in slowing the progression of liver fibrosis in patients with T2DM because statins can reduce hepatic expression of tumor necrosis factor-α (TNF-α), interleukin 6 (IL-6) and transforming growth factor-β (TGF-β) [13]. However a previous study also showed that statins were not associated with the progression of nonalcoholic fatty liver disease [24]. Aspirin was also found to have some effect on decreasing aspartate aminotransferase platelet ratio index(APRI) scores in patients with chronic liver disease. However, the association between antidepressant use and liver fibrosis in patients with T2DM has been unexplored until now.

In our present study, we included SSRIs, TCAs and SNRIs, which are the main types of prescriptions for patients with depressive disorder. Previous studies have reported that TCAs may play an antifibrotic role in liver disease, and may exert their antifibrotic effect in hepatic stellate cells through inhibition of the sphingomyelinase pathway [25]. Moreover, TCAs reduced NAFLD-induced hepatic steatosis, suggesting that TCAs may reduce NASH by regulating endoplasmic reticulum (ER) stress, lysosomal membrane permeabilization, and autophagy [26]. However, some recent evidence has suggested that TCAs are associated with increased weight gain and insulin resistance among patients with depression [27, 28]. The complex relationship between TCAs, T2DM and NAFLD should be noted. On the other hand, there is an increasing evidence that long-term SSRI use is associated with an increasing incidence of hepatic lipid accumulation [29]. The potential mechanisms are not fully understood. Some emerging evidence suggests that serotonin production, which can act via the 2A serotonin receptor (HTR2A) to upregulate the expression of lipogenic proteins and increase hepatic steatosis, in the periphery may be instrumental to the pathophysiology of NAFLD [30, 31]. SSRIs including fluoxetine have been shown to increase serotonin synthesis in previous studies [29]. In our present study, 18.8% of diabetic patients received antidepressant drugs, which mainly included SSRIs, TCAs and SNRIs. We found that antidepressant drugs were not associated with the degree of hepatic fibrosis on liver biopsy, but also on VCTE.

Our study has several strengths and limitations. To our knowledge, our study was the first to assess the association between antidepressant use and VCTE-based liver fibrosis in patients with type 2 diabetes. Moreover, we had a large sample size and decided to include both sexes and all age groups. However, as an observational and cross-sectional study design, our study also has several limitations. First, as a cross-sectional analysis, temporal trends of antidepressant use and liver fibrosis cannot be extrapolated. Second, in the database, we do not have data concerning the duration of antidepressant use, and prospective studies should be conducted in the future to examine the association of liver fibrosis and antidepressant use.

## Conclusion

In conclusion, in this cross-sectional study, we found that antidepressant drugs were not associated with liver fibrosis or cirrhosis in patients with type 2 diabetes in a nationwide population. Further prospective studies or RCTs should be conducted to validate this finding.

## Data Availability

All the data are available in NHAHES database.
